# Validity and reliability of the English version of the Diabetic Foot Self-Care Questionnaire: a cross-cultural adaptation

**DOI:** 10.3389/fpubh.2023.1326439

**Published:** 2024-01-24

**Authors:** María Ruiz-Muñoz, Raúl Fernández-Torres, Cynthia Formosa, Alfred Gatt, Gabriel Gijón-Noguerón, Emmanuel Navarro-Flores, Manuel González-Sánchez

**Affiliations:** ^1^Department of Nursing and Podiatry, Faculty of Health Sciences, University of Malaga, Malaga, Spain; ^2^Department of Podiatry, Faculty of Health Sciences, University of Malta, Msida, Malta; ^3^Department of Nursing, Faculty of Nursing and Podiatry, University of Valencia, Valencia, Spain; ^4^Department of Physiotherapy, Faculty of Health Sciences, University of Malaga, Malaga, Spain

**Keywords:** diabetic foot, chronic complications, patient-reported outcome, questionnaire, self-care, assessment

## Abstract

**Introduction:**

The objective of this study was to carry out the cross-cultural adaptation and validation of the Diabetic Foot Self-Care Questionnaire into the English language, broadening the applicability of this patient-reported outcome measure and improving the monitoring of patients with diabetic foot disease.

**Methods:**

The validation study into English was conducted in two phases: cross-cultural adaptation and psychometric validation study. Short Form-12 Version 2, EuroQoL-5D and Foot Function Index were used to analyze the criterion validity. Item response, internal consistency, standard error of measurement, minimal detectable change and construct validity were calculated in the validation phase.

**Results:**

An English version of the questionnaire (DFSQ-UMA-En) was successfully obtained. A total of *n* = 193 participants were tested to confirm the validity and reliability of the questionnaire. Internal consistency values ranged from very good to excellent (Cronbach’s *α* =0.889–0.981), and reliability was excellent (ICC = 0.854–0.959). Standard error measurement value was =2.543. Criterion validity ranged from *r* = 0.429 to *r* = 0.844. For construct validity, Kaiser-Meyer-Olkin test was =0.752.

**Conclusion:**

DFSQ-UMA-En is a valid and reliable tool with good readability and comprehension features. This questionnaire addresses foot self-care behaviors in patients with diabetic foot disease, standing out as essential for early diagnosis and prevention strategies in clinical and research settings.

## 1 Introduction

According to the International Working Group on the Diabetic Foot, diabetic foot disease (DFD) is defined as the infection, ulceration, or destruction of tissues of the foot of an individual diagnosed with diabetes mellitus (DM), coexisting with neuropathy and/or peripheral arterial disease in the lower limbs (1). It is the chronic complication of DM with the highest mortality rate, most frequently caused by amputation of the lower limbs (2).

Epidemiology data and costs due to hospitalization are worsening over the years, with incidence and prevalence being higher in low-income areas (3, 4). In this context of resource scarcity, the best prevention strategies arise from the early diagnosis of DFD based on the implementation of assessment tools with high accuracy, availability, and applicability.

Assessment tools aimed at diagnosis can be classified according to the source of the given outcome: biomarkers, objective clinical outcome measures, and clinician-reported or patient-reported outcome measures (CROMs and PROMs, respectively) (5). The latter are capable of tracking changes in clinical symptoms over time, improving the quality of care, and enhancing disease control, in addition to their easy distribution and low cost (6).

Recent systematic reviews have concluded that PROMs lack availability and psychometric quality for patients with DFD. The Diabetic Foot Self-Care Questionnaire (DFSQ-UMA) was identified as the best option for assessing foot self-care (7–9). The evaluation of self-care is essential in chronic diseases, since higher levels of self-care have been associated with better health outcomes, including decreased hospitalization, costs, and mortality (10). DFSQ-UMA is currently available in the Spanish, French and Arabic languages (11–13).

Global data from 2022 confirms that the English language is the largest according to the number of speakers, and the third largest language according to the number of native speakers (about 373 million native speakers) (14). Highly populated countries with high income inequality and low gross domestic product *per capita*, such as India, Nigeria and South Africa, are examples of English-speaking countries (15).

The objective of this study is to make the cross-cultural adaptation and validation of the DFSQ-UMA into the English language, broadening the applicability of this PROM and allowing for the improvement of the monitoring of DFD patients.

## 2 Materials and methods

### 2.1 Study design

The validation study of DFSQ-UMA into English was conducted in two different phases. The first phase consisted in the translation and cross-cultural adaptation of the DSFQ-UMA from its original version into English (DFSQ-UMA-En). The second stage consisted in a validation study and analysis of the psychometric properties of the DFSQ-UMA-En.

The participants of this study were recruited from the Diabetic Foot Unit of the Birkirkara Health Center (Birkirkara, Malta), from 1st October 2022 to 30th January 2023, and based on the following inclusion criteria: aged 18 or older, diagnosed with diabetic foot disease, and no history of major surgery on the lower limbs. On the other hand, the study excluded those participants who did not autonomously understand the questions due to cognitive impairment, as well as those who were not native English speakers or sufficiently proficient in English. Participants who left any of the questions unanswered were also excluded.

### 2.2 Ethical considerations

This study was conducted in accordance with the recommendations of the Declaration of Helsinki, following the ethical principles for research involving human subjects, and the data were handled in accordance with Organic Law 3/2018 of December 5th, regarding the Protection of Personal Data and the Guarantee of Digital Rights. All participants provided their informed consent to participate in the study. Additionally, the Ethics Committee of the University of Malta approved the execution of this study with protocol number 4113_26032020.

### 2.3 Diabetic Foot Self-care Questionnaire of the University of Malaga

The DFSQ-UMA is a 16-item questionnaire that was designed to analyze self-care practices in patients with DFD. The DFSQ-UMA consists of three specific subcategories of foot self-care: self-care (assessed by questions 1–7), self-management and self-examination (assessed by questions 8–11), and footwear and socks (assessed by questions 12–16). Each question is scored on a Likert scale from 0 to 4, for a maximum total score of 64. The obtained score is then weighted on a 0-to-100 scale for better result comprehension, where higher values indicate poorer self-care (11, 12).

### 2.4 Translation and cross-cultural adaptation

Guidelines from the International Test Commission along with recommendations from the current scientific literature were followed to ensure that the conceptual and terminological adaptation of the questions in the DFSQ-UMA was carried out correctly (16).

The process of translating and cross-culturally adapting the DFSQ-UMA into the English version was carried out in four steps:

- Translation of the DFSQ-UMA from the original Spanish version into English: this translation was performed by two native and independent translators who were blinded to each other. The translations were compared to create the preliminary version of the DFSQ-UMA-En.
- A back-translation into the original language was performed by two independent translators, who were native Spanish speakers.
- The preliminary version was reviewed by an expert committee consisting of *n* = 7 researchers, who discussed any discrepancies between versions.
- A pilot test was conducted with *n* = 25 subjects using the obtained version before reaching the final version.

**Figure 1:**
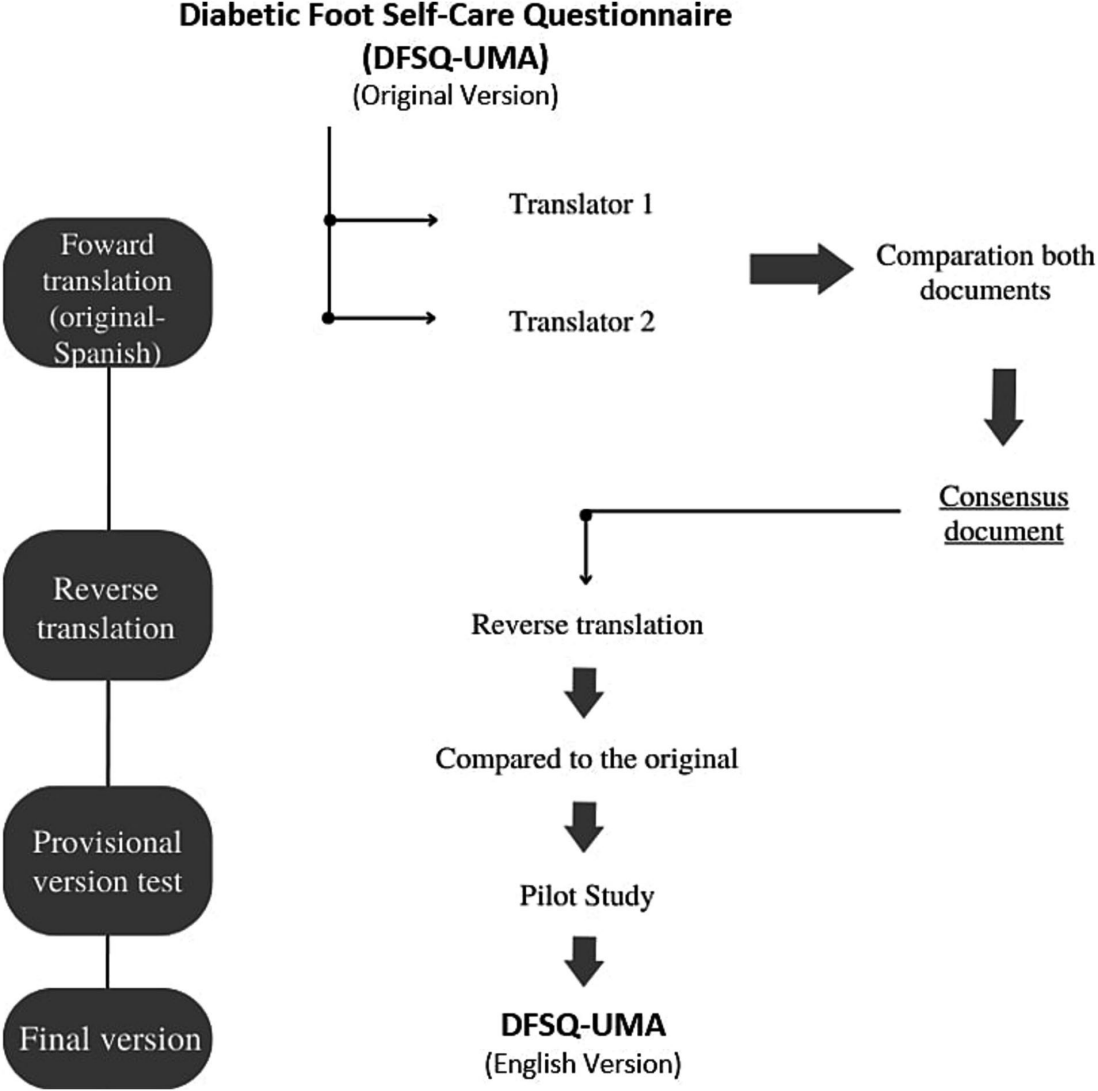
Flowchart of the cross-cultural adaptation of the DFSQ-UMA—English version.

Figure 1 presents a schematic summary of the entire process undertaken in the translation and cross-cultural adaptation of the DFSQ-UMA-En.

### 2.5 Criterion validity

To perform criterion validity tests, the following questionnaires were used:

*Short Form-12 Version 2 (SF-12-v2)*: This questionnaire consists of 12 items designed to assess well-being and functional capacity. In addition to the results obtained from version 1 (Physical and Mental Health Status), it yields 8 new dimensions (in alphabetical order): bodily pain, physical functioning, social functioning, emotional role, physical role, mental health, general health, and vitality. The score for each component ranges from 0 to 100, where a higher score indicates better health status. This questionnaire has shown reliability values ranging from 0.60 to 0.78, with internal consistency >0.8 (17).

EuroQoL-5D: this questionnaire is composed of 5 questions that assess the quality of life of individuals through 5 different dimensions: mobility, self-care, usual activities, pain/discomfort, and anxiety/depression. Each dimension is assessed using three different levels: 1 - no problems; 2 - slight or moderate problems; 3 - severe problems or incapacity. The combination of all questions with possible responses allows describing 243 potential health states, which are weighted based on the responses provided to derive a score on health status and quality of life. Additionally, EuroQol-5D is complemented by a visual analogue scale (VAS) that assesses the patient’s perceived quality of life, where higher values indicate better quality of life. The reliability of EuroQol is indicated by an ICC = 0.7 (18).

*Foot Function Index (FFI)*: this self-administered questionnaire consists of 23 questions aimed at evaluating functional capacity in the foot by assessing stiffness, social functioning, difficulty in movement, activity level, and pain. The reliability for this questionnaire ranges from 0.69 to 0.87, while internal consistency ranges from 0.73 to 0.96 (19).

### 2.6 Data collection

Following the recommendations of the COSMIN guidelines, all participants initially completed the English version of the DFSQ-UMA-En, EuroQoL-5D, SF-12, and FFI. Subsequently, to perform the analysis of internal validity, reliability, and the calculation of consistency levels, the participants filled out the DSFQ-UMA-En again 5 days after the first completion. Two blinded researchers were responsible for data collection and analysis.

### 2.7 Data analysis

A descriptive analysis of the sample was conducted in two different approaches, depending on the variable under consideration. Firstly, the frequency of questions regarding participants’ educational level, gender, and pharmacological treatment was described. Then, a descriptive analysis of the sample was performed by calculating the mean and standard deviation for age and duration of DM diagnosis, as well as for the chosen measurement instruments: DFSQ-UMA-En, SF-12-v2, EuroQoL-5D, and FFI. Subsequently, a distribution analysis of the sample was conducted using the Kolmogorov–Smirnov test. The ceiling/floor effects were analyzed, which were considered to be present when at least 15% of participants achieved the maximum or minimum value on the DFSQ-UMA-En in their responses.

To calculate item response, the intraclass correlation coefficient (ICC) was used, while the internal consistency of the measures was calculated using Cronbach’s α. Both measures were classified using the following scale: excellent: ≥0.80; good: 0.60–0.80; moderate: 0.40–0.60; and poor: ≤0.40 (20).

The standard error of measurement (SEM) and the minimal detectable change at 90% confidence level (MDC90) were calculated. To calculate SEM, the formula SEM = s√1 – r was used, where “s” represents the test score’s standard deviation, and “r” was Pearson’s correlation coefficient. For MDC90, the formula MDC90 = SEM × √2 × 1.65 was utilized.

For the analysis of the structure and construct validity, the maximum likelihood extraction method was performed. Since this is a cross-cultural adaptation of the DFSQ-UMA into the English version, the authors decided to maintain the original questionnaire’s structure. For this reason, factor extraction was forced into three factors.

For criterion validity, the SF-12-v2, EuroQoL-5D, and FFI questionnaires were used. A correlation analysis was conducted between the DFSQ-UMA-En and these questionnaires through the calculation of Pearson’s coefficient. The results were structured according to the following scale: *r* ≥ 0.75 (strong); 0.50 ≤ *r* ≤ 0.74 (moderate); and *r* ≤ 0.49 (poor) (21). The structure and validity of the construct was analyzed from the extraction by maximum likelihood (EML). To maintain the original structure of the DFSQ-UMA, a 3-factor forced model was performed. In addition, to perform the EMV, the requirement of a minimum of 10 subjects per item was satisfied (minimum number = 90 – subjects measured = 243).

Criterion validity was calculated by analyzing the degree of correlation between the DFSQ-UMA and the Spanish versions of the questionnaires: EuroQoL-5D (22), FFI (23), SF-12-v2 (24). Pearson’s correlation coefficient was structured according to the following scale: r ≤ 0.49 (poor), 0.50 ≤ r ≤ 0.74 (moderate), r ≥ 0.75 (strong). To perform the statistical analysis of this study, the SPSS software V.23.0 (Armonk, NY, United States) was used.

In order to conduct the statistical analysis, the literature recommends having a final sample size equal to or greater than 10 subjects for each item included in the questionnaire. Therefore, to carry out the cross-cultural adaptation and validation study of the DFSQ-UMA-En, 160 subjects would be required. This study was conducted with *n* = 193 subjects (25). To perform the Exploratory Factor Analysis (EFA), the requirement of a minimum of 10 subjects per item was met (minimum number = 90 – items measured = 243). The statistical software IBM SPSS Statistics V.23 (Armonk, NY, United States) was utilized for the statistical analyzes in this study.

## 3 Results

The translated and adapted English version of the DFSQ-UMA (DFSQ-UMA-En) is provided in Supplementary material. Table 1 presents the descriptive characteristics of the sample, showing the results as a function of participant gender, education level, and pharmacological treatment. The description is based on the frequency of the responses obtained. It is observed that 51.8% of the surveyed participants were males, with the predominant education level being primary education, followed by higher education, accounting for 45.8 and 35.9% of participants, respectively. The most frequently used pharmacological treatment was oral medication, with a total of 136 patients (70.5%).

**Table 1:** Descriptive characteristics of the sample and frequencies.

		Frequency	Percentage	Cumulative percentage
Sex	Male	100	51.8	51.8
	Female	93	48.2	100
Education level	Primary education	90	45.8	45.8
	Secondary education	67	35.9	81.7
	Higher education	30	15.4	97.1
	Other	1	0.7	97.8
	None	5	2.2	100.0
Pharmacological treatment	Oral	136	70.5	70.5
	Insulin	20	10.4	80.8
	Both	29	15.0	95.9
	None	8	4.1	100.0
*N*	193			

Table 2 presents the descriptive characteristics of the sample for those variables where the mean and standard deviation of the results were calculated. These variables include the age of the participants and the number of years since they were diagnosed with diabetes. Furthermore, the results of all outcome variables, namely DFSQ-UMA-En, SF12-v2, EuroQoL-5D, and FFI, both the total values and those obtained in all the subscales composing these tools, are included. In the analysis of the floor/ceiling effect, it was observed that 7 participants achieved the minimum score, while 9 reached the maximum score, accounting for 3.6 and 4.7% of the participants, respectively. Based on the observed results, it is considered that the floor/ceiling effect is not relevant in DFSQ-UMA-En. The average time to complete DFSQ-UMA-En was 4.21 min.

**Table 2:** Descriptive characteristics of the sample based on mean and SD.

		Min	Max	Mean	SD
Age (years)		42	90	65.29	10.56
DM duration (years)		0	53	15.83	11.62
DFSQ-UMA-En	Total	0	100	65.99	12.70
	Self-care	15	35	28.15	5.46
	Self-assessment	7	20	16.28	2.41
	Footwear and socks	12	24	19.91	2.76
FFI	Pain	5	22	9.65	4.42
	Stiffness	6	18	9.95	2.48
	Difficulty	11	39	20.18	6.37
	Activity	2	12	3.90	1.64
	Social	6	18	7.96	2.22
	TOTAL	33	91	51.64	13.23
SF-12	Physical Function	22.11	51.81	32.39	9.72
	Role Physical	20.32	30.98	23.72	4.25
	Bodily Pain	16.68	26.87	17.92	3.31
	General Health	18.87	44.74	29.90	3.36
	Vitality	27.62	40.87	36.67	2.75
	Social Functioning	16.18	36.37	20.83	7.76
	Role Emotional	11.35	22.53	20.73	2.86
	Mental Health	21.87	34.06	32.04	3.45
	Physical Component State	17.43	31.20	20.99	3.72
	Mental Component State	29.21	53.51	39.02	7.72
EuroQol_VAS		0.230	1.000	0.73	0.20
EuroQol_5D		19.00	100.00	76.34	16.49
N		193			

The internal consistency analysis of the DFSQ-UMA-En yielded Cronbach’s α values ranging from 0.889 (self-exploration) to 0.981 (self-care). In addition, item response results showed ICC values ranging from 0.854 (Item 9) to 0.959 (Item 14). For further details, please refer to Table 3. Furthermore, the observed SEM values were 2.543, while the MDC90 was 5.933.

**Table 3:** Internal consistency and reliability.

Cronbach’s Alpha	Total	0.928
	Self-care	0.981
	Self-assessment	0.889
	Footwear and socks	0.897
ICC (Item responses)	0.854 (Item 9)–0.959 (Item 14)	

**Figure 2:**
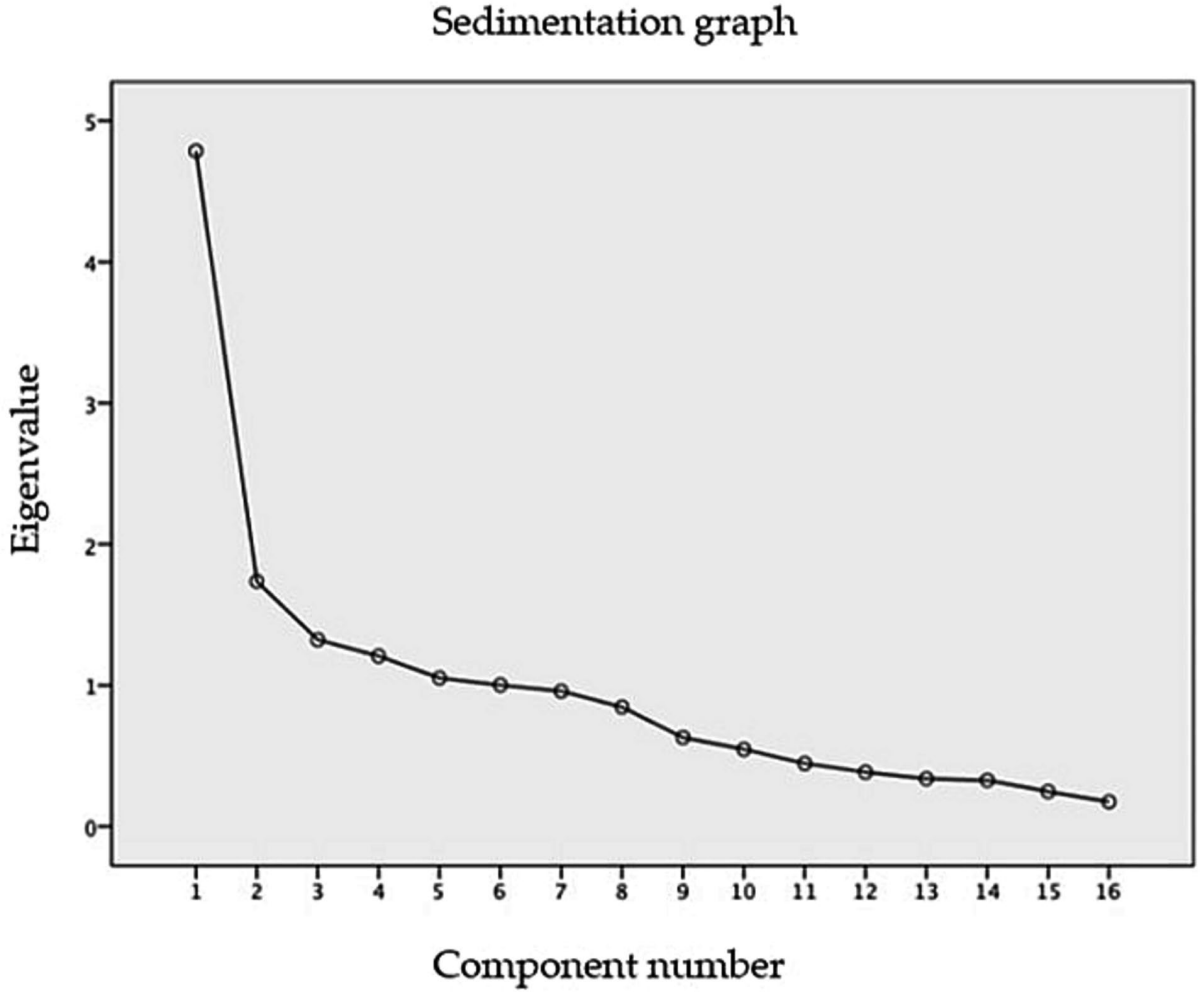
Sedimentation plot of the 16 items of the English version of DFSQ-UMA (DFSQ-UMA-En).

Regarding construct validity assessment, the Kaiser-Meyer-Olkin (KMO) test yielded a value of 0.752, indicating statistically significant Bartlett’s sphericity test results (*p* < 0.001), with 120 degrees of freedom and a Chi-square value of 1070.326. When forcing the extraction to three factors, they collectively explained 49.035% of the variance (see Table 4). Table 5 displays the loadings of each item on the three extracted factors, while Figure 2 illustrates the screen plot of all the items that comprised the DFSQ-UMA-En.

**Table 4:** Eigenvalues and variance explained by items from the DFSQ-UMA En.

Component	Eigenvalues			Squared charge extraction sums		
	Total	Variance (%)	Cumulative %	Total	Variance (%)	Cumulative %
1	4.787	29.919	29.919	4.787	29.919	29.919
2	1.736	10.853	40.772	1.736	10.853	40.772
3	1.322	8.264	49.035	1.322	8.264	49.035
4	1.207	7.544	56.580			
5	1.051	6.571	63.151			
6	1.002	6.261	69.412			
7	0.958	5.988	75.400			
8	0.845	5.282	80.682			
9	0.630	3.937	84.620			
10	0.545	3.409	88.029			
11	0.446	2.788	90.816			
12	0.384	2.403	93.219			
13	0.338	2.113	95.332			
14	0.327	2.042	97.374			
15	0.246	1.541	98.914			
16	0.174	1.086	100.000			

**Table 5:** Matrix of components from DFSQ-UMA-En.

	Factor 1	Factor 2	Factor 3
DFSQ-UMA-En_1	0.731	−0.221	−0.440
DFSQ-UMA-En _2	0.744	−0.142	−0.286
DFSQ-UMA-En _3	0.746	−0.145	−0.219
DFSQ-UMA-En_4	0.732	0.082	−0.111
DFSQ-UMA-En_5	0.646	0.248	−0.170
DFSQ-UMA-En_6	0.507	0.229	0.015
DFSQ-UMA-En_7	0.718	0.021	0.224
DFSQ-UMA-En_8	0.491	−0.425	0.346
DFSQ-UMA-En_9	0.236	−0.469	−0.221
DFSQ-UMA-En_10	0.450	−0.491	0.348
DFSQ-UMA-En_11	0.287	0.262	0.469
DFSQ-UMA-En_12	0.493	0.182	0.175
DFSQ-UMA-En_13	0.440	0.494	0.399
DFSQ-UMA-En_14	0.384	0.445	−0.222
DFSQ-UMA-En_15	0.032	0.583	−0.277
DFSQ-UMA-En_16	0.498	−0.015	0.287

For criterion validity assessment, the SF12-V2, EuroQoL-5D, and FFI questionnaires, as well as various subcategories of different questionnaires, were utilized. Table 6 displays the correlation values of the DFSQ-UMA-En, along with its different subcategories. Both the overall score of the DFSQ-UMA-En and the self-care subcategory exhibited higher correlation levels with all reference instruments for criterion validity compared to those observed in the self-exploration and socks/shoes subcategories. For a more in-depth analysis of the correlation results, please refer to Table 6.

**Table 6:** Correlation between DFSQ-UMA-En and the instruments used to analyze criterion validity.

		DFSQ-UMA-En			
		Self-care	Self-assessment	Footwear and socks	Total
SF-12	Physical Function	0.557**	0.256**	0.354**	0.706**
	Role Physical	0.628**	0.202**	0.446**	0.802**
	Bodily Pain	0.425**	0.074	0.312**	0.475**
	General Health	0.401**	0.306**	0.208**	0.429**
	Vitality	0.646**	0.354**	0.443**	0.780**
	Social Functioning	0.594**	0.146*	0.422**	0.683**
	Role Emotional	0.486**	0.302**	0.302**	0.696**
	Mental Health	0.565**	0.325**	0.369**	0.727**
	Physical Component State	0.629**	0.186**	0.438**	0.676**
	Mental Component state	0.631**	0.215**	0.414**	0.801**
EuroQol VAS		0.858**	0.364**	0.482**	0.844**
EuroQol 5D		0.924**	0.372**	0.431**	0.744**
FFI	Pain	0.637**	0.215**	0.384**	0.646**
	Stiffness	0.667**	0.189**	0.356**	0.645**
	Difficulty	0.719**	0.253**	0.419**	0.642**
	Activity	0.537**	0.127	0.322**	0.565**
	Social	0.640**	0.181*	0.415**	0.685**
	Total	0.858**	0.275**	0.506**	0.831**

Significance of Pearson correlation coefficient *p ≤ 0.01; **p ≤ 0.001.

## 4 Discussion

The main objective of this study was to perform the translation and the cross-cultural adaptation of DFSQ-UMA into the English language, as this is a specific questionnaire for the assessment of foot self-care in patients with DFD. The readability and comprehension features regarding the items of the new English version were satisfactory, as well as in terms of psychometric properties.

### 4.1 Translation and cross-cultural adaptation of DFSQ-UMA into DFSQ-UMA-En

In addition to the original version of the DFSQ-UMA in the Spanish language, this questionnaire has been previously translated into other languages, such as Arabic (12), Persian (26), Turkish (27), and French (13). The process of translation and validation of this questionnaire in English followed the current recommendations in the scientific literature (28). This included the use of native translators who were external to the field of study to facilitate the comprehension of the translated version while ensuring that the terminology used remained consistent to maintain the sense and meaning of the questions as in the original version. This translation and cultural adaptation will, therefore, provide all clinical professionals and researchers in English-speaking regions with a tool for assessing the self-management of patients with DFD.

Furthermore, the potential results obtained using this questionnaire in English can be compared with results measured in other versions of the same questionnaire, allowing for cross-cultural and cross-linguistic research in the field of diabetic foot care and management.

### 4.2 Construct validity

During the construct validity analysis, the researchers decided to maintain the structure of the original questionnaire, as it is a translated version (11). The analysis proved that the DFSQ-UMA had a three-factor structure. The three factors extracted frm the DFSQ-UMA-En explained a total variance of 49.035%. Other versions have also performed exploratory factor analyzes, extracting three factors. However, the explained variance value varies between 48.1% in the Arabic version (slightly higher than that in the French version) and 60.88% in the original version. Therefore, the explained variance falls within the range of values observed in the other versions that have been analyzed (12, 13, 26, 27).

Kaiser-Meyer-Olkin test showed a value of 0.752 in the English version, which is lower than the values of 0.866 and 0.872 observed in the Turkish and Persian versions, respectively, as well as the 0.89 observed in the original and French versions. However, if the usual criteria for factor extraction had been strictly followed, i.e., >10% variance, eigenvalue >1.0, and the inflection point of the screen plot, it seems that the DFSQ-UMA-En would have a two-factor structure.

### 4.3 Internal consistency and test–retest validity

The DFSQ-UMA-En questionnaire demonstrated internal consistency, as indicated by Cronbach’s *α* = 0.928. The sub-scales showed values ranging from 0.889 (self-exploration) to 0.981 (self-care) (Table 3). These results agree with those observed in the Arabic version, where internal consistency ranged from 0.887 to 0.983, as well as the French version (Cronbach’s *α*: 0.911–0.925). However, they appear to be higher than those observed in the original version (Cronbach’s *α*: 0.89), calculated only for the total score of the questionnaire, and in the Persian and Turkish versions, where Cronbach’s α values ranged from 0.750 to 0.884 and 0.771 to 0.880, respectively.

Furthermore, item response results showed ICC values ranging from 0.854 (Item 9) to 0.959 (Item 14) (Table 3). The observed results are consistent with those of the original version (ICC: 0.89–0.92) and the Arabic version (ICC: 0.841–0.956). However, the observed values were higher than those reported in the Turkish version (ICC: 0.32–0.69) and the French version (ICC: 0.48).

### 4.4 Criterion validity

The FFI, SF12-V2, and EuroQoL-5D questionnaires, along with their subdimensions, were used for criterion validity. Correlation levels of the total score of the DFSQ-UMA-En with each category of the SF12-v2 are generally good or excellent, except for “Body pain” and “General health” subcategories. On the other hand, the strongest correlation observed with the EuroQoL-5D questionnaire was not achieved with the total score of the DFSQ-UMA-En (ranging from 0.744 to 0.844), but rather with the “Self-care” subcategory, which showed correlation values of 0.858 and 0.924.

This might indicate that, while the DFSQ-UMA-En reliably and validly assesses the quality of life of patients, the scores of the other subcategories also allow for the evaluation of complementary aspects of the subject. Similarly, the correlation observed with the FFI questionnaire, where correlation values are good or excellent, can be interpreted in a similar manner.

In summary, the DFSQ-UMA-En appears to be a valuable tool for assessing the quality of life of patients with DFD, and its subcategories provide insights into various aspects of their well-being and self-care. The correlations with other established questionnaires indicate the questionnaire’s validity and its ability to complement existing assessment tools. When comparing the DFSQ-UMA-En with other versions of the DFSQ-UMA, it becomes apparent that only two of the published versions (the original version and the French version) conducted criterion validity analyzes. However, it is important to note that the reference instruments used for this analysis in those versions differ from those used in the DFSQ-UMA-En. In the original version (11), criterion validity was assessed by correlating the questionnaire with HbA1c levels and blood sugar levels, while the French version used HbA1c levels (13), resulting in correlation values of *r* = 0.15 (HbA1c) and *r* = 0.226 (glucose) in the original version and *r* = 0.17 (HbA1c) in the French version. Consequently, the results observed in these different versions cannot be directly compared with those used in the validation of the DFSQ-UMA-En.

Comparing criterion validity across different versions of a questionnaire can be challenging when reference instruments and validation methodologies differ. Researchers should carefully consider the specific context and goals of their research when selecting a version of the questionnaire, in order to ensure that it aligns with their objectives and the characteristics of their study population.

For the assessment of criterion validity, the FFI, EuroQol, and SF12-v2 questionnaires were chosen, not matching the questionnaires selected for the original validation study in the Spanish language (11). This choice was due to a broader availability of valid and reliable questionnaires in the English language that fulfill more accurately the purposes of DFSQ-UMA.

Other questionnaires with good psychometric properties such as the Diabetes Self-Management Questionnaire (29) and Summary of Diabetes Self-Care Activities (30) do not address foot care. The exclusion of foot self-care in DM patients ignores the high mortality rate of DFD and the costs it generates on public healthcare systems (2, 4), thus the presence of items regarding foot health should be considered in the development of future PROMs.

### 4.5 Future research implications

The adaptation of questionnaires into other languages allows comparing the results obtained in different settings that may use the same instrument. Therefore, this facilitates the development of common intervention, assessment, and monitoring strategies. However, the adaptation process must adhere to recommendations found in the literature, which implies questionnaire translation, cross-cultural adaptation, and the evaluation of psychometric characteristics. These steps ensure that the developed versions maintain content equivalence and serve as clear, reliable, and assessable tools (31).

From this approach, the DFSQ-UMA is a specially designed tool for assessing self-care practices among patients with DFD. The accurate evaluation of this aspect is crucial, since numerous interventions proposed in the literature to improve cost-effectiveness rely on patient education. Specific aspects reflected in the DFSQ-UMA, such as self-care, self-assessment, and footwear/sock management, are essential. Having a tool explicitly designed to assess this therapeutic aspect is pivotal for effective patient monitoring.

This study demonstrates that the DFSQ-UMA-En is an optimal tool for assessing and monitoring self-care practices among English-speaking population with DFD. This will enable clinical professionals and researchers to conduct future investigations. There is one questionnaire similar enough to DFSQ-UMA in the scientific literature, i.e., the Diabetes Foot-Self Care Behavior Scale (DFSBS) (32), which is a 7-item questionnaire with good psychometric properties available in the Chinese, Farsi and German languages (33, 34). However, a recent systematic review on PROMs in DFD patients recommends DFSQ-UMA over DFSBS, as the former meets the current recommendations to a greater extent compared to the latter (7, 35).

### 4.6 Strengths and weaknesses

One of the main strengths of this study is the adaptation of the DFSQ-UMA into English, which is the language that serves as the backbone for the dissemination of clinical and scientific knowledge worldwide. English is also the most widely spoken non-native language globally, thus this adaptation facilitates its use in English-speaking population and research, increasing the visibility of this valuable tool. This, in turn, may encourage the translation and cross-cultural adaptation of this instrument into other languages to allow for result comparisons across different population groups.

Moreover, this validation study exceeded the minimum number recommended by the literature for the validation of assessment tools based on the number of items. In this case, 160 individuals were required, and the study was conducted on 193 patients (25).

This study has some limitations that should be taken into consideration. The English version of DFSQ-UMA was adapted using a sample of type 2 DM patients within a specific age range, excluding those under 18 years of age. Subsequent research could consider specific population profiles due to psycholinguistics differences, such as patients with associated comorbidities (36), as well as the design of studies to analyze psychometric variables related to longitudinal studies, such as responsiveness.

## 5 Conclusion

DFSQ-UMA-En is a valid and reliable tool with good readability and comprehension features. The Cross-cultural adaptation and validation of DFSQ-UMA into the English language were successful. This questionnaire addresses foot self-care behaviors in DFD patients, standing out as essential for early diagnosis and prevention strategies in clinical and research settings.

## Data availability statement

The data presented in this paper will be available from the corresponding author upon reasonable request.

## Ethics statement

The studies involving humans were approved by University of Malta Research Ethics Committee. The studies were conducted in accordance with the local legislation and institutional requirements. The participants provided their written informed consent to participate in this study.

## Author contributions

MR-M: Conceptualization, Data curation, Investigation, Project administration, Validation, Writing – review & editing. RF-T: Investigation, Software, Validation, Writing – original draft. CF: Formal analysis, Funding acquisition, Writing – review & editing. AG: Formal analysis, Supervision, Writing – review & editing. GG-N: Resources, Visualization, Writing – review & editing. EN-F: Supervision, Writing – original draft. MG-S: Conceptualization, Methodology, Writing – original draft, Writing – review & editing.
