# Supplementary material for: Validity and reliability of the English version of the Diabetic Foot Self-Care Questionnaire: a cross-cultural adaptation
Source: Front Public Health. 2024 Jan 24;11:1326439. doi: 10.3389/fpubh.2023.1326439 (PMC10851747; doi:10.3389/fpubh.2023.1326439)
Supplement: Supplementary file 1 [file Data_Sheet_1.pdf]

The diabetic foot self-care questionnaire (English version) (DFSQ-UMA-En)

We would like to know how you practice self-care of your feet. Please choose the option that best suits your habits. Please answer all the questions.

1. In general, do you check your feet yourself?

a. Several times a day.
b. Once a day.
c. 2 or 3 times a week.
d. Once a week.
e. I don't examine my feet.

2. Do you check yourself for the presence of wounds or the condition of the skin of your feet?

a. Once a day.
b. 2 or 3 times a week.
c. Once a week.
d. Sometimes.
e. I don't examine my feet.

3. Do you inspect the condition of your nails? (Do not answer if you have undergone amputation of the toes).

a. Everyday.
b. Once a week.
c. Once every 2 weeks.
d. Once a month.
e. I don't examine my feet.

4. How important is it for you the frequency of personal foot care?

a. I consider it very important, and I review and take care of it personally daily.
b. I think it's quite important, I check them daily, but I don't pay attention to personal care.
c. I think it's important and I take care of them personally, but I don't check them daily.
d. I consider it of little importance, sometimes I take care of my feet and check them.
e. I think it's not important, I don't check my feet or take care of them.

5. Regarding the recommendations on how to take care of your feet...

a. I have received information and take care of my feet.
b. I have received information, but I don't take care of my feet.
c. I have not received information on how to care for them, but I try to pay attention to them.
d. I have received information on how to take care of them, but I don't pay attention to them.
e. I have not received information and I do not know how to take care of them.

6. How do you practice self-care for dry skin and calluses?

a. I use moisturizer and a foot file.
b. I use only a foot file.
c. I use blades or scalpels.
d. I use some corn preparations.
e. I don't pay attention to my feet.

7. To dry your feet ...

a. I use a towel only for the feet and dry the sole of the foot and between the toes.
b. I use a towel only for the feet and dry the sole of the foot.
c. I use the same towel as for the body and to dry the plantar foot and between the toes.
d. I let them air dry.
e. I cannot dry them.

8. Is it difficult for you to find comfortable shoes because of your feet?

a. It's not hard to find.
b. A little hard to find.
c. Pretty hard to find.
d. Very hard to find.
e. Impossible to find.

9. How often do you cut or treat your toenails? (Do not respond if you have undergone amputation of the toes).

a. I do it with a frequency of between 1 to 15 days.
b. I do it with a frequency of between 15 to 30 days.
c. I do it with a frequency of between 1 to 2 months.
d. I do it with a frequency greater than 2 months.
e. I don’t do it.

10. Do you find it difficult to dry your feet after showering? (Do not respond if you have undergone amputation of the feet).

a. It isn’t hard to do it.
b. A little hard to do it.
c. Pretty hard to do it.
d. Very hard to do it.
e. Impossible to do it.

11. Is it difficult for you to find adequate socks due to your feet?

a. I have no difficulties.
b. I have some difficulties.
c. I have quite a bit of difficulties.
d. I have many difficulties.
e. It is impossible to find suitable socks.

12. Regarding conventional footwear, before using it...

a. I check that there are no objects inside, that it is without seams, spacious and with laces.
b. I make sure that it is wide, and I check the interior.
c. I note if they are comfortable and flexible but without trying them.
d. It is important that design is attractive.
e. I do not give importance to footwear.

13. Regarding socks…

a. I check that they are made of natural fibers and without seams.
b. I check that they are not tight against the leg and the thigh.
c. I use synthetic socks.
d. I do not care about the material.
e. I don't wear socks or socks.

14. Regarding new footwear…

a. I make sure that it is comfortable and, if not, I change it.
b. If is not comfortable I change it with another more comfortable shoes.
c. I try to adapt it using it little by little.
d. I don't care if it's uncomfortable.
e. I don't pay attention to new footwear.

15. Regarding summer footwear, when it is very hot…

a. I wear shoes suitable for heat (breathable).
b. Alternate open and closed shoe.
c. I wear flip flops or sandals.
d. I walk barefoot frequently.
e. I don't pay attention and it is not important for me.

16. To warm your feet...

a. I use socks made of wool or natural fibers.
b. I use heat sources such as stoves or radiators.
c. I use hot water bottles.
d. I take hot baths.
e. I don't care what I use.
